# Collection media and delayed freezing effects on microbial composition of human stool

**DOI:** 10.1186/s40168-015-0092-7

**Published:** 2015-08-12

**Authors:** Roberto Flores, Jianxin Shi, Guoqin Yu, Bing Ma, Jacques Ravel, James J. Goedert, Rashmi Sinha

**Affiliations:** Nutritional Science Research Group, Division of Cancer Prevention, National Cancer Institute, National Institutes of Health, 9609 Medical Center Dr. RM5E554-MSC9788, Bethesda, MD 20892 USA; Biostatistics Branch, Division of Cancer Epidemiology and Genetics, NCI/NIH, Bethesda, MD USA; Infections and Immunoepidemiology Branch DCEG/NCI/NIH, Bethesda, MD USA; Institute of Genome Sciences, University of Maryland School of Medicine, Baltimore, MD USA; Nutritional Epidemiology Branch, DCEG/NCI/NIH, Bethesda, MD USA

## Abstract

**Background:**

Different bacteria in stool have markedly varied growth and survival when stored at ambient temperature. It is paramount to develop optimal biostabilization of stool samples during collection and assess long-term storage for clinical specimens and epidemiological microbiome studies. We evaluated the effect of collection media and delayed freezing up to 7 days on microbial composition. Ten participants collected triplicate stool samples each into no media as well as RNAlater® with and without kanamycin or ciprofloxacin. For each set of conditions, triplicate samples were frozen on dry ice immediately (time = 0) or frozen at −80 °C after 3-days and 7-days incubation at 25 °C. Microbiota metrics were estimated from Illumina MiSeq sequences of 16S rRNA gene fragments (V3–V4 region). Intraclass correlation coefficients (ICC) across triplicates, collection media, and incubation time were estimated for taxonomy and alpha and beta diversity metrics.

**Results:**

RNAlater® alone yielded the highest ICCs for diversity metrics at time = 0 [ICC median 0.935 (range 0.89–0.97)], but ICCs varied greatly (range 0.44–1.0) for taxa with relative abundances <1 %. The 3- and 7-day freezing delays were generally associated with stable beta diversity for all three media conditions. Freezing delay caused increased variance for Shannon index (median ICC 0.77) and especially for observed species abundance (median ICC 0.47). Variance in observed species abundance and in phylogenetic distance whole tree was similarly increased with a 7-day delay. Antibiotics did not mitigate variance. No media had inferior ICCs at time 0 and differed markedly from any media in microbiome composition (e.g., *P* = 0.01 for relative abundance of Bacteroidetes).

**Conclusion:**

Bacterial community composition was stable for 7 days at room temperature in RNAlater® alone. RNAlater® provides some stability for beta diversity analyses, but analyses of rare taxa will be inaccurate if specimens are not frozen immediately. RNAlater® could be used as collection media with minimal change in the microbiota composition.

## Background

Relationships between the gut bacteria and health are not new, but an emerging concept is that altered functions of the bacterial community contribute to disease development collectively, rather than through the action of specific pathogenic members. Next generation sequencing approaches have provided powerful tools to study associations of the human microbiome with disease. Interesting associations of microbiota and disease have been reported [1–3], but their etiologic significance has not been assessed in well-powered case–control or prospective epidemiological studies. To move the field of human microbiome research forward both for clinical purposes and in epidemiological research, validated methods are needed for collecting specimens that represent, as closely as possible, the true *in vivo* parameters and to understand any technical variation that can be introduced. For large population-based microbiome studies, specimen collection methods must be acceptable to participants and, most importantly, tolerant of suboptimal field conditions. If optimal collection and storage conditions (i.e. immediate freezing and storage at −80 °C) are not possible, systematic bias can be introduced in preprocessing steps [4]. Therefore, it is imperative to minimize possible artifacts by developing and validating collection methods than can be easily implementable for both clinical uses and for large field-based epidemiologic studies.

Analysis of microbial diversity in human specimens poses important challenges, especially and particularly for field epidemiological studies where a cold chain cannot always be maintained or assured from sample collection to freezer storage. Specimens collected in the field may often spend various amounts of time at room temperature, followed by shipment on frozen gel packs (4 °C) or dry ice to a central laboratory for processing or storage. Fecal samples may not be representative of the whole gastrointestinal (GI) tract or specific loci within the GI, but a recent study showed that it is the relative abundance of taxa that differs rather than the lack of representation in stool as compared to mucosa-associated microbiota [5]. For screening purposes stool could be used to detect and quantify putative bacterial species as biomarkers of microbiota associated carcinogenesis as in the case of colorectal cancer [6–8].

Recent efforts to systematically evaluate and standardize post collection analysis of the microbiota will undoubtedly homogenize protocols and facilitate comparison among studies (www.mbqc.org). However, few studies have evaluated the pre-analytical steps focusing on stability of the microbiome in stool samples collected under field conditions. Very few reports have focused on the impact of preservation medium, time and temperature on the microbial community structure and other microbiota metrics of alpha and beta diversity [9]. RNAlater® has been suggested as the preservative of choice to conserve the stability of nucleic acids, both DNA and RNA, in tissue and other biospecimens [10–14]. However, the sufficiency of RNAlater® alone to prevent differential growth of bacteria during typical delays in field studies is unknown. Addition of antibiotics that prevents either RNA transcription or protein translation may improve biostabilization. Previous reports have yielded inconsistent results for the effects of room temperature storage on DNA and RNA stability for microbial analysis [15–19] As the microbiome field is advancing from descriptive to longitudinal or prospective studies, it is important to systematically evaluate the collection methods and the media used to biopreserve the microbiome integrity in stool. In this report, we describe a systematic evaluation of the effects of preservation media and storage conditions on the composition of the fecal microbiota as analyzed by 16S rRNA gene profiling (V3-V4 region) using Illumina MiSeq sequencing. The objective of this study was to evaluate RNAlater as a biopreservative for large, population-based studies; a biopreservative for human microbiome analyses of stool samples that remain unfrozen for as long as seven days.

## Results and discussion

### Reproducibility of microbiota metrics with no collection media and RNAlater-based collection media

**Fig. 1:**
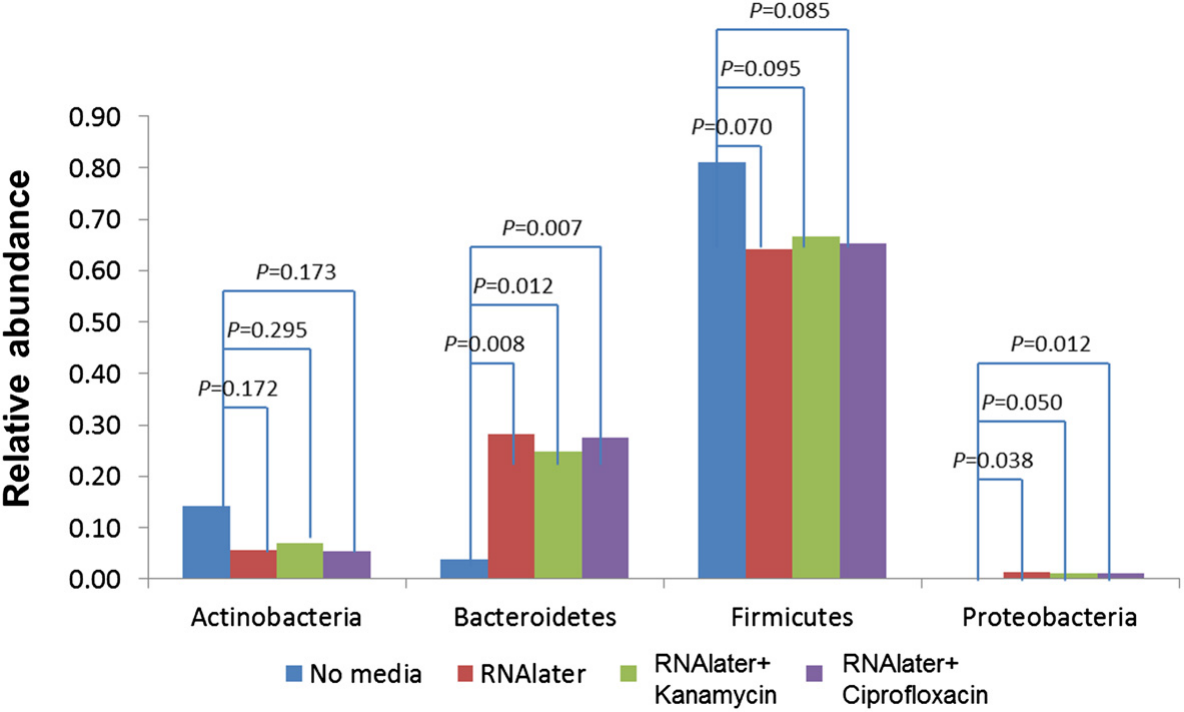
Comparison of major phyla in fecal samples stored in different media at baseline

We first compared the microbial composition of stool replicates collected without collection media to those collected with different media at time zero. As shown in Fig. 1, compared to specimens collected in RNAlater®-based media, specimens collected without media had significantly different microbial composition, with a marked reduction in relative abundance of Bacteroidetes, a smaller reduction of Proteobacteria, and a compensatory increase in Actinobacteria and Firmicutes.

Compared to media conditions, particularly RNAlater® alone, the no media condition also had lower ICCs for reproducibility of relative abundance for Bacteroidetes (ICC 0.71), alpha diversity estimates (mean of four estimates, ICC 0.865 versus 0.935 for RNAlater® alone), and for some of the PCoA scores (Table 1). Based on these differences in relative abundance and reproducibility, we restricted the rest of our analysis to conditions using media.

**Table 1:** Technical reproducibility, estimated as intraclass correlation (ICC) coefficient and UniFrac distance-based *R* ^2^, under different conditions and time points

	Relative^a^ abundance	Time = 0				Time = 3 days			Time = 7 days		
		No media	RNAlater alone	RNAlater + Kanamycin	RNAlater + Ciprofloxacin	RNAlater alone	RNAlater + Kanamycin	RNAlater + Ciprofloxacin	RNAlater alone	RNAlater + Kanamycin	RNAlater + Ciprofloxacin
Actinobacteria	2.1 %	0.98	0.96	0.97	0.92	0.96	0.98	0.89	0.95	0.96	0.99
Bacteroidetes	14.6 %	0.71	0.93	0.84	0.91	0.93	0.88	0.85	0.94	0.97	0.86
Firmicutes	75.1 %	0.97	0.93	0.82	0.89	0.94	0.89	0.84	0.95	0.97	0.87
Proteobacteria	0.6 %	0.95	0.94	0.83	0.32	0.98	0.83	0.90	0.94	0.90	0.88
PD_WT		0.87	0.95	0.87	0.82	0.75	0.47	0.69	0.48	0.72	0.40
Chao1		0.81	0.91	0.74	0.72	0.66	0.25	0.55	0.30	0.59	0.17
Observed_species		0.83	0.92	0.82	0.74	0.74	0.31	0.65	0.46	0.65	0.16
Shannon		0.95	0.96	0.92	0.80	0.89	0.70	0.87	0.76	0.83	0.52
Unweighted.PCoA1		0.96	0.97	0.94	0.95	0.92	0.81	0.93	0.91	0.94	0.83
Unweighted.PCoA2		0.80	0.96	0.87	0.75	0.87	0.61	0.86	0.75	0.84	0.38
Unweighted.PCoA3		0.82	0.89	0.89	0.86	0.89	0.76	0.66	0.86	0.93	0.77
Weighted.PCoA1		0.74	0.95	0.83	0.94	0.92	0.92	0.87	0.90	0.97	0.88
Weighted.PCoA2		0.97	0.97	0.96	0.97	0.96	0.96	0.94	0.97	0.97	0.98
Weighted.PCoA3		0.90	0.91	0.88	0.90	0.91	0.76	0.87	0.95	0.95	0.96
Unweighted UniFrac		0.70	0.75	0.76	0.67	0.79	0.64	0.72	0.70	0.74	0.66
Weighted UniFrac		0.91	0.97	0.92	0.80	0.97	0.89	0.97	0.93	0.91	0.81

^a^Median of the relative abundance across all subjects

### Reproducibility of microbiota metrics with different collection media at time zero

We first sought to identify differences at time zero among the three media (RNAlater® alone, RNAlater® with kanamycin, or with ciprofloxacin) by calculating ICCs for 14 microbiota metrics across triplicate samples from the 10 subjects. These metrics included relative abundances of the major phyla (Proteobacteria, Bacteroidetes, Firmicutes, Actinobacteria), four alpha-diversity metrics (Shannon, Chao-1, PD_WT, richness), and beta diversity represented by PCoA scores (Table 1). For all 14 microbiome measurements, ICCs were significantly larger than 0 (*P*≤1.0×10^−3^). As shown in Table 1, RNAlater® alone yielded the highest ICCs in all but one of the 14 metrics. However, with only 10 subjects, none of the pairwise comparisons was statistically significant. Mean values of the PCoA scores closely resembled beta diversity estimates based on weighted UniFrac (0.92 for RNAlater alone) and unweighted UniFrac (0.76 for RNAlater alone).

**Fig. 2:**
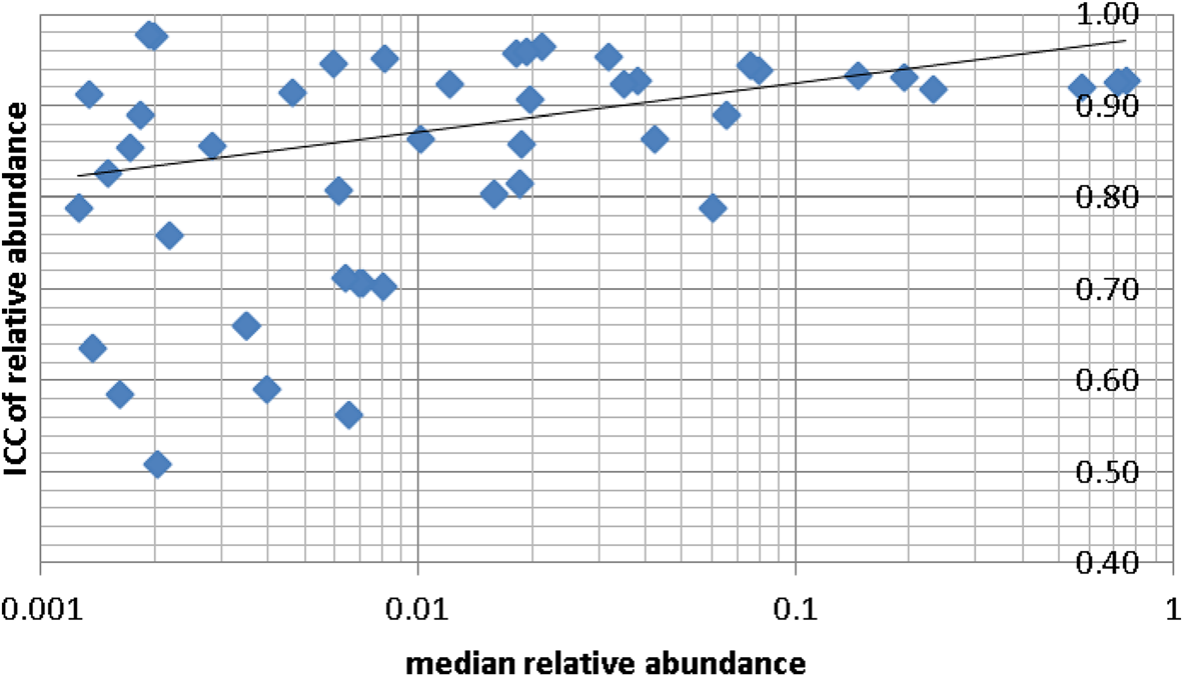
The relative abundance of more common taxon tend to be more reproducible under condition RNAlater at time 0. The figure is based on taxa with median relative abundance greater than 0.1 % across all samples. Each *diamond* represents a taxon. The line was fitted by linear regression using all data points

We also investigated whether ICCs differed across taxa by their relative abundances (excluding taxa with relative abundance <0.1 %). We restricted this analysis to the RNAlater® alone condition, because it delivered the highest ICCs. ICCs were high (interquartile range 0.92-0.93) for taxa with relative abundances >8 % and varied ICCs (interquartile range 0.82-0.96) for taxa with intermediate relative abundances (1-8 %). A linear regression model of the mean relative abundance (log scale) and the ICC of relative abundance showed that taxa with relative abundances <1 % were highly significantly correlated with varied ICCs (interquartile range 0.73-0.96) (Fig. 2, *P*_trend_=0.0007).

### Stability of microbiota metrics with delayed freezing

For each media condition and all 14 microbiota metrics, we compared immediate freezing (time=0) to incubation for 3 days (time=3) or 7 days (time=7) at room temperature prior to freezing. Across triplicates and subjects, we calculated ICCs for time=3 vs 0, and for time=7 vs 0 with each media (Table 2).

**Table 2:** Stability, estimated as intraclass correlation (ICC) coefficient and UniFrac distance-based *R* ^2^, between two time points

		ICC					
	Relative^a^ abundance	Time 3 days vs. Time 0				Time 7 days vs. Time 0	
		RNAlater alone	RNAlater + Kanamycin	RNAlater + Ciprofloxacin	RNAlater alone	RNAlater + Kanamycin	RNAlater + Ciprofloxacin
p__Actinobacteria	2.10 %	0.96	0.98	0.97	0.98	0.92	0.95
p__Bacteroidetes	14.60 %	0.76	0.82	0.81	0.70	0.79	0.69
p__Firmicutes	75.10 %	0.73	0.81	0.77	0.68	0.77	0.69
p__Proteobacteria	0.60 %	0.60	0.77	0.54	0.64	0.88	0.62
PD_WT		0.64	0.47	0.56	0.85	0.62	0.64
Chao1		0.40	0.03	0.12	0.76	0.44	0.34
Observed_species		0.53	0.23	0.36	0.78	0.53	0.41
Shannon		0.77	0.77	0.76	0.88	0.80	0.69
Unweighted.PCoA1		0.90	0.88	0.91	0.97	0.91	0.92
Unweighted.PCoA2		0.65	0.46	0.58	0.88	0.78	0.64
Unweighted.PCoA3		0.79	0.78	0.64	0.94	0.93	0.80
Weighted.PCoA1		0.80	0.83	0.86	0.61	0.80	0.79
Weighted.PCoA2		0.93	0.95	0.97	0.96	0.96	0.97
Weighted.PCoA3		0.85	0.91	0.82	0.90	0.86	0.87
Unweighted UniFrac		0.70	0.71	0.70	0.76	0.76	0.71
Weighted UniFrac		0.78	0.86	0.87	0.75	0.86	0.80

^a^Median of the relative abundance across all subjects

For time=3 vs 0, taxonomy ICCs (relative abundances by phylum) were higher with RNAlater® with kanamycin, especially for relatively rare Proteobacteria taxa (ICC_kanamycin_ 0.77, vs ICC_RNAlater®_0.60 and ICC_ciprofloxacin_ 0.54, Table 2). For Shannon index estimate of alpha diversity, ICC was 0.76-0.77 for all three media. For the other three alpha diversity estimates, ICCs were very low, especially ICC_kanamycin_ (0.03-0.47) and ICC_ciprofloxacin_ (0.12-0.56). For unweighted beta diversity, the first principle coordinate had high ICCs (0.88-0.91) with all three media. For weighted beta diversity, the first three principal coordinates had high ICCs with all three media (ICC_RNAlater®_0.80-0.93; ICC_kanamycin_ 0.83-0.95; ICC_ciprofloxacin_ 0.82-0.97, Table 2).

**Fig. 3:**
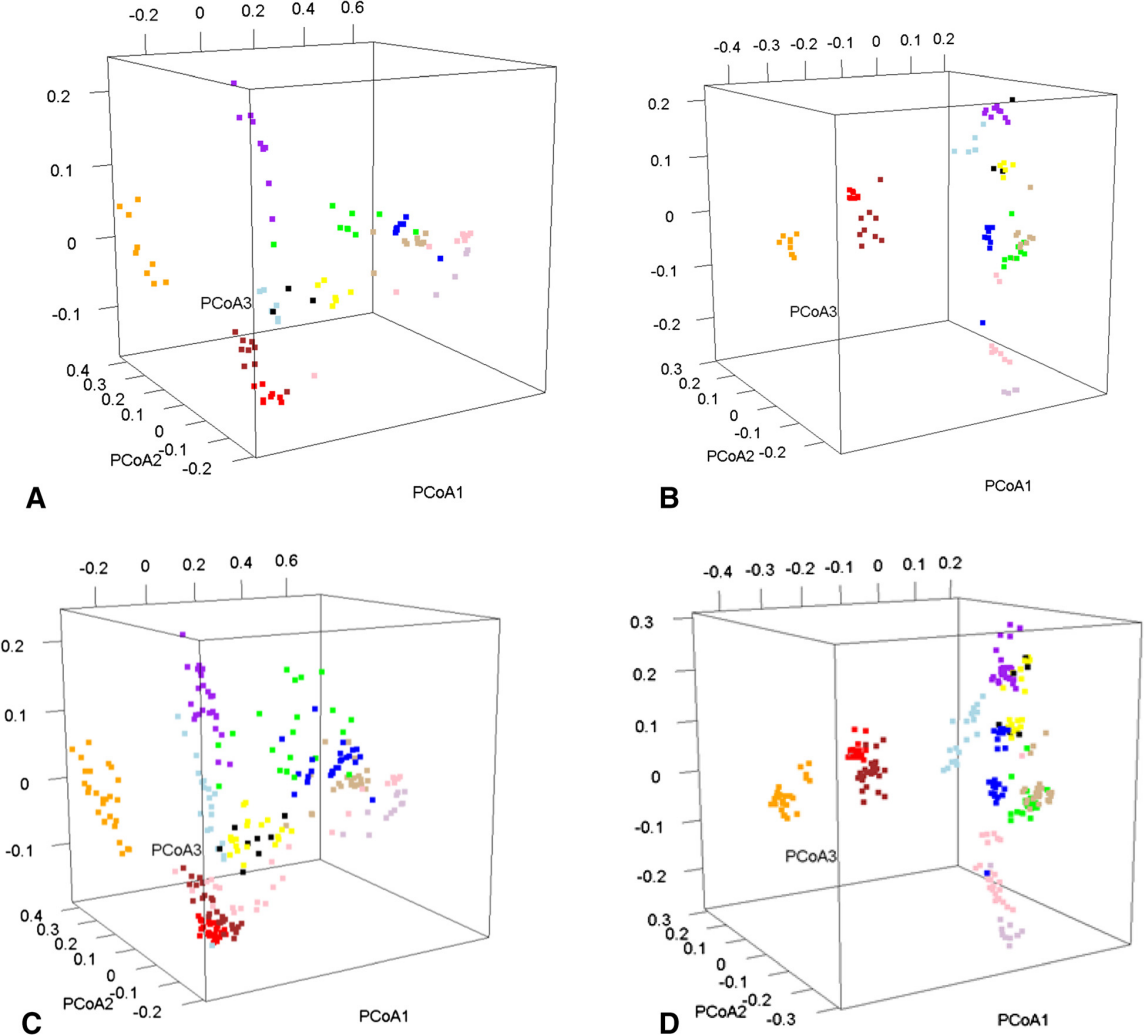
Principal coordinate analysis of fecal microbiota to evaluate structure reproducibility under different sampling and storage conditions. Samples from each of the ten subjects are presented with a different color. **a** Time 0, weighted beta diversity with all media conditions. **b** Time 0, unweighted beta diversity at time 0 with all media conditions. **c** Weighted beta diversity across all media conditions and three time points. Variation explained was 54.1, 11.6, and 5.7 % for PCoA1, 2, and 3, respectively. **d** Unweighted beta diversity across all media conditions and three time points. Variation explained was 14.3, 11.1, and 7.6 % for PCoA1, 2, and 3, respectively

For time=7 vs 0, ICCs with the three media generally followed the same pattern as for time=3 vs 0 (Table 2). For example, taxonomy ICCs (relative abundances by phylum) were higher with RNAlater® with kanamycin (e.g., Proteobacteria taxa ICC_kanamycin_ 0.88, vs ICC_RNAlater®_ 0.64 and ICC_ciprofloxacin_ 0.62). Other than Shannon index, alpha-diversity ICCs were much higher with RNAlater® alone (0.76-0.85) than RNAlater® with kanamycin (0.44-0.62) or ciprofloxacin (0.34-0.64). Except for a few principal coordinates, both unweighted and weighted beta diversity ICCs for time=7 vs 0 were high with all three media. Mean values of the PCoA scores closely resembled beta diversity estimates based on weighted UniFrac (0.92 for RNAlater alone) and unweighted UniFrac (0.76 for RNAlater alone). The strong clustering of weighted beta diversity for each of the 10 subjects, incorporating differences over time and all three media, is illustrated in Fig. 3.

The importance of the human gastrointestinal (GI) microbiota in health and its possible association with disease is becoming more evident [20–23]. However, there is high heterogeneity in specimen collection methods in the literature and no consensus on methods to optimally stabilize specimens for microbial composition analysis. Our study found that human fecal specimens collected in RNAlater® yield highly reproducible microbiome composition (beta diversity) estimates, even if they were held up to 7 days at room temperature (25 °C) prior to freezing. Despite this, detection and relative abundances of rare taxa were altered by delayed freezing, irrespective of media, which highlights the potential for bias if specimens are not handled optimally.

To enhance stabilization of the microbiota ex vivo, we conducted preliminary experiments (not presented) of RNAlater® without and with individual and combined antibiotics. Of all the conditions tested, two antibiotics, kanamycin (through inhibition of protein synthesis) and ciprofloxacin (through inhibition of DNA replication) showed promising results when used at bactericidal concentration (data not shown). The current study built on these findings by supplementing RNAlater® with a bactericidal dose (300 μg/ml) of more than 10-fold the minimal inhibitory concentration of these two antibiotics, which we compared to RNAlater® alone. We found that adding antibiotics to RNAlater® did not yield more stable microbiota metrics in feces left at room temperature for 3 or 7 days. In fact, RNAlater® alone had the highest ICC values for all major phyla for day 3 and was consistent over time (day 7). Irrespective of delayed freezing, the high reproducibility of independent replicates observed for overall composition of the bacterial communities (PCoA plots) suggests that relatively few replicates are needed with RNAlater® alone as the collection media. This finding has major implications with regard to large epidemiologic studies since the cost of collecting and storing multiple aliquots could be substantially reduced.

Surprisingly, we observed statistically significant differences in the microbial composition of the same stool sample in aliquots collected in RNAlater®-based collection media compared to no media. All samples underwent the same extraction method, but our results indicate a major reduction in relative abundances of Bacteroides in stool samples collected without collection media. The no media condition also had inferior ICCs, which was severe for reproducibility of relative abundance for Bacteroidetes (ICC 0.71), and also notable for alpha diversity estimates and some beta diversity estimates (PCoA scores). It is possible that the thawing process of stool stored without collection media affect the integrity of members of the phylum Bacteroides. Perhaps such stool samples stored without collection media needs a special digestion step after thawing, or requires the immediate inhibition of potential nucleases that are liberated during thawing.

Other studies have determined the effect of storage parameters with stool samples used for microbial genomic analysis. However, these studies focused on measuring the quality and quantity of extracted DNA [24], the effect of room temperature on stool with no preservation media [25], the robustness of pyrosequencing using stool samples stored in different conditions and DNA extraction methods [15], or the effect of temperature incubation (including freezing) on measures of bacterial communities in sputum [26]. Lauber et al. reported on the stability of stool biospecimens for microbiome analysis using 16SrRNA gene sequencing. In that study, the stool samples were left at room temperature without storage media for up to two weeks and found the condition satisfactory for microbial community analysis even after this prolonged period [9]. However, because no storage media were tested in that study, it is not possible to evaluate if the differences we observed were stable over time. In addition, while we did not test the stability over time without storage media, our results also show stability of microbial community composition for up to 7 days in RNAlater®. Different mechanisms may contribute to stability. In Lauber et al., the stool was aliquoted using cotton swabs with no preservation media and only DNA was analyzed. The cotton swab may have acted as a desiccant for the stool, thus preventing further bacterial growth during the incubation period. In our study, the RNAlater® solution, being a liquid media, may diffuse more easily to the core of the specimen given proper mixing, and is believed to act as both DNA and RNA stabilizer and inactivate nucleases when specimens are thawed before extraction. In addition, RNAlater® is design to enable measures of RNA transcripts and thus increase its utility for future epidemiologic studies.

Of interest, a recent report by Cardona et al. suggests that DNA and RNA from stool samples may degrade at room temperature even if collected in RNAlater® affecting the composition of the microbiota for meta-genomic and -transcriptomic analyses [17]. It is unclear, however, if the observed degradation of RNA in samples collected with RNAlater® also corresponds to changes in DNA integrity and microbial classification since stool samples collected with RNAlater® were not used for microbial taxonomic analysis. Our results showed that RNAlater® alone provides sufficient stability for taxonomic analyses of bacterial DNA from stool specimens even if left at room temperature for up to 7 days.

The major limitations of our study are its small size, with only 10 participants, and exclusive focus on how delayed freezing and selected media affect the fecal microbiome. Nonetheless, these issues are critically important for large epidemiologic field studies. These were rigorously evaluated with state-of-the-art microbiome laboratory and analysis methods. Without question, more research is needed on other factors that can affect statistical power and bias, including handling, storing, and thawing of specimens, microbial cell lysis, nucleic acid extraction, robustness of sequencing platforms, and classification and quantification of microbial taxa. The inclusion of mock communities that are site-specific would be necessary in future studies of the microbiome, especially those using clinical specimens, to validate the methodology in the analysis of microbial communities. As reported in a recent study by Hang and colleagues, such mock communities have been developed and evaluated for thermal stability with the aim to be used as reference for microbiome studies [27].

## Conclusions

The technological advancements in sequencing over the last 20 years have revolutionized our conceptual framework of microbiota and host interactions. Assessment of bacterial composition and specific bacterial functions can be used as a powerful tool to understand their role in health and disease risk. As a step to help move the microbiome field to epidemiological studies, herein we evaluated parameters of microbiome stability and effects of delayed freezing and collection media on bacterial community structure based on 16S rRNA gene sequences. Our data suggest that RNAlater® alone effectively preserves the composition of the fecal microbial community for up to 7 days at room temperature, implying that it could be used for population-based studies in field settings. The high ICC observed suggests that fewer replicates can be collected thus saving costs and storage space. Independent validation is needed, as is expansion to understand extreme environments and possible artifactual effects on microbial gene expression.

## Methods

### Study participants

Ten healthy volunteers (8 male and 2 female) were aged between 34–61 [mean 44.2]. Five participants had taken an antibiotic or other medication within one year. Four participants were taking probiotics at the time of the study. Following face-to-face instructions and signed informed consent, participants were provided written and illustrated instructions and a toilet-attached pouch (Protocult, Rochester, MN), from which they collected 30 samples of an early or mid-morning stool. After specimen collection, they completed a brief self-administered questionnaire on demographics, broad dietary categories, ease-of-use of the devices, and factors potentially related to the gut microbiota. The study was reviewed and approved by the National Cancer Institute Special Studies Institutional Review Board (protocol 10CN107).

### Stool specimen collection

Participants were recruited to assess the stability and reproducibility of microbial measures in self-collected fecal specimens following a protocol as previously described [28]. Participants used Sarstedt (Nümbrecht, Germany) fecal collection devices containing solutions as described below to collect 30 separate samples from various parts of a single stool. The fecal vials were either frozen on dry ice immediately or were incubated at 25 °C (equivalent of room temperature) for the times described below, following which they were all stored at −80 °C until used for DNA extraction.

### Design, conduct and analysis of the effects of room temperature and storage media on detection and classification of bacterial taxa in stool specimens

A summary of the experimental conditions are shown in Table 3. Briefly, each of the ten participants collected 30 aliquots from a single stool, each containing ~0.5-1 g of feces. Four conditions were used. Three aliquots were collected in no media; nine aliquots in 5ml of RNAlater® (Ambion, Austin, TX); nine aliquots in 5ml of RNAlater® containing 300 μg/ml kanamycin (SIGMA B5264, St Louis, MO); and nine aliquots in 5ml of RNAlater® containing 300 μg/ml ciprofloxacin (SIGMA 17850). Three aliquots for each condition were immediately frozen on dry ice (provided to participants) and the rest of aliquots were kept at room temperature, then all samples were brought to the laboratory. After arrival in the lab, the frozen aliquots were stored at −80 °C and the remaining aliquots were incubated at room temperature (25 °C). The incubation times were 72 hours (3 days) and 168 hours (7 days) at which time they were stored at −80 °C. Once frozen, all aliquots were kept at −80 °C until used for DNA extraction.

**Table 3:** Collection media type and room temperature incubation times evaluated in the study

Media tested	Incubation time 0	Incubation time 1	Incubation time 2
		(72 h at 25 °C)	(168 h at 25 °C)
No media	3 Aliquots	–	–
RNAlater®	3 Aliquots	3 Aliquots	3 Aliquots
RNAlater® + Kan^a^	3 Aliquots	3 Aliquots	3 Aliquots
RNAlater® + Cipro^b^	3 Aliquots	3 Aliquots	3 Aliquots

Study participants (*n* = 10) sampled aliquots (*n* = 30) from a single stool which they stored under different conditions. Subsamples were collected either without collection media or three different RNAlater-based media and frozen immediately after collection (time 0) or stored by two time periods (72 and 168 h) at 25 °C before freezing on dry ice and stored at −80 °C

^a^RNAlater® supplemented with 300 μg/ml Kanamycin

^b^RNAlater® supplemented with 300 μg/ml Ciprofloxacin

### Fecal DNA extraction

Genomic DNA from stool samples was extracted with a modification of the stool QIAamp DNA Stool mini kit (QIAGEN, Valencia, CA). Briefly, 300 mg of feces was mixed with 350 μL of lysis buffer composed of 0.05 M potassium phosphate buffer containing 50 μL lyzosyme (10 mg/mL), 6 μL of mutanolysin (25,000 U/ml; Sigma-Aldrich) and 3 μL of lysostaphin (4,000 U/mL in sodium acetate; Sigma-Aldrich). The mixture was incubated for 1 hour at 37 °C, then 10 μL proteinase K (20 mg/ml), 100 μL 10 % SDS, and 20 μL RNase A (20 mg/ml) were added, and the mixture was incubated for 1h at 55 °C. Microbial cells were lysed by mechanical disruption (bead beating) using a FastPrep instrument (MP Biomedicals, Solon, OH) set at 6.0 m/s for 30 sec. The lysate was processed using the QIAsymphony SP protocol Pathogen complex 400 (Qiagen, Gaithesburg, MD) according to the manufacturer’s recommendation. The DNA was eluted into 100 μL of storage buffer [QIAsymphony reagent buffer AVE (0.04 % sodium azide), Qiagen], pH 8.0. PCR inhibitors were removed from the extracted DNA using the Zymo-Spin IV Spin Filter column according to the manufacturer’s recommendations (Irvine, CA). DNA was quantified by Quant-iT PicoGreen (Molecular Probes, Inc., Eugene, OR) in a SpectraMax M5 microplate reader (Molecular Devices, Sunnyvale, CA).

### PCR and Illumina MiSeq sequencing of the V3-V4 regions of 16S rRNA genes

A region of approximately 469 bp encompassing the V3 and V4 hypervariable regions of the 16S rRNA gene was targeted for sequencing. This region provides ample information for taxonomic classification of microbial communities and was used by the Human Microbiome Project [29]. Fusion dual barcoded primers 319F (5’ ACTCCTACGGGAGGCAGCAG – 3’) and 806R (5’ – GGACTACHVGGGTWTCTAAT- 3’) were used to amplify the V3-V4 region of bacterial 16S rRNA genes [30]. The amplicons were pooled in equimolar concentration and sequenced on an Illumina MiSeq Instrument using the 250 bp paired-end protocol.

### Analysis of 16S rRNA (V3-V4 region) sequence data and classification of Operational Taxonomic Units (OTUs)

**Fig. 4:**
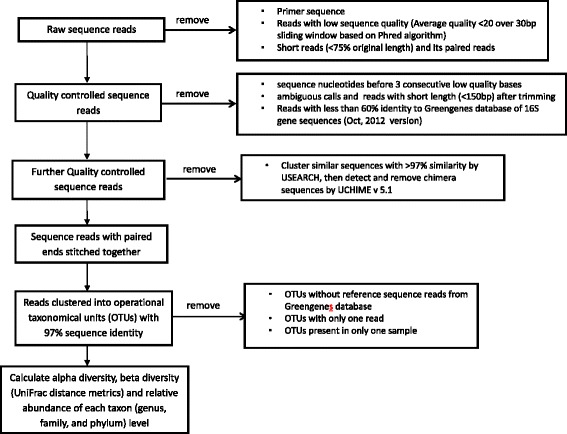
Flow diagram for the analysis of 16S rRNA sequence data, classification of operational taxonomic units, and generation of diversity metrics

Quantitative Insights Into Microbial Ecology (QIIME), an open source software package [31] was used for sequence analysis (see Fig. 4). Sequence reads were filtered using the QIIME pipeline with the following criteria to optimize the quality and integrity of the data: i) removal of primer sequence, ii) truncation of reads not having an average quality of 20 over a 30 bp sliding window based on the phred algorithm [32, 33], iii) removal of trimmed reads having less than 75 % of their original length, and iv) removal of the paired reads that were discarded for having less than 75 % original length. QIIME (version 1.6.0) [31] was used for all further sequence processing steps, including quality trimming and demultiplexing. Quality trimming in QIIME was performed using the following criteria: 1) no ambiguous base calls, 2) truncate sequence before 3 consecutive low quality bases and re-evaluate for length, 3) minimum sequence length of 150 bp after trimming, and 4) remove sequences with less than 60 % identity to a pre-built Greengenes database of 16S rRNA gene sequences (Oct, 2012 version) [34]. Further data processing included clustering similar sequences with less than 3 % dissimilarity using UCLUST [35] and *de novo* chimera detection and removal in UCHIME v5.1 [36]. Paired reads were stitched together with “N” between each sequence and processed as one sequence in the analysis. The sequence reads were then clustered at 97 % nucleotide sequence identity in QIIME. A closed-reference Operational Taxonomic Unit (OTU) picking protocol with USEARCH against the Greengenes database was employed. Sequence reads that did not match the Greengenes database were excluded from further analyses. Of the 1,869,502 amplicon sequences processed, 94.4 % (1,765,159) hit a reference sequence at greater than or equal to 97 % sequence identity.

### Microbiota metrics of alpha and beta diversity

We evaluated 14 microbiota metrics across triplicate samples from the 10 subjects. These metrics included relative abundances of the major phyla (Proteobacteria, Bacteroidetes, Firmicutes, Actinobacteria), four alpha-diversity metrics (Shannon, Chao-1, PD_WT, richness), and beta diversity represented by PCoA scores [37]. Briefly, richness, which is the total number of unique OTUs, does not take relative abundance of OTUs into account. Chao1 is bias-corrected for singleton OTUs [38]. Shannon index is a conservative alpha diversity estimate that adjusts for the relative abundance (proportion) of each taxon. Shannon index is defined as (negative) the sum over taxa of the product of the relative abundance of each taxon times the natural logarithm of its relative abundance. That is, where H is Shannon index and pi is the proportion of total species represented by species i, H=−Σ[(pi)*ln(pi)] [39]. PD_WT is a measure of alpha diversity that reflects phylogenetic divergence among OTUs within a sample. In order to compare microbiota diversity between individuals at the same sequence depth, a random sample of 5000 OTUs was drawn without replacement from each sample 20 times.

Weighted and unweighted UniFrac distances between samples, which measure the pairwise phylogenetic distances between microbial communities, were calculated in QIIME by using the existing tree from the Greengenes database [40]. Weighted UniFrac distance accounts for the relative abundance of each taxon in the communities while unweighted UniFrac distance does not. From the UniFrac distance matrix, the top three vectors produced from a principal coordinate analysis (PCoA) were used for downstream reproducibility analysis.

### Statistical analyses

For each condition at time zero, we calculated the mean of relative abundance or alpha diversity for each condition across the samples from the 10 subjects. We performed t-tests, without correcting for multiple comparisons, of whether no media was significantly different from each of the media conditions (RNAlater® with or without antibiotic) for relative abundances of taxa in the four major Phyla.

Next, we used intraclass correlation coefficient (ICC) to quantify the reproducibility of relative abundances of taxa, alpha diversity metrics and PCoA scores based on weighted and unweighted UniFRac distance matrix. The ICC is defined as *σ*_*b*_^2^/(*σ*_*b*_^2^ + *σ*_*ε*_^2^) with *σ*_*b*_^2^ representing between subject variability and *σ*_*ε*_^2^ representing within subject variability (at each time point). We calculated the ICCs using the R package “ICC” estimated on a mixed effect model [41]. For each ICC, we tested if ICC=0, i.e. the measures are random across technical replicates, using permutations. A high value of ICC (between 0 and 1) indicates a high reproducibility of the measurement across technical replicates. We tested whether the ICCs were different among the three media conditions using permutations. We also quantified the percentage of overall microbiota variability explained by subjects by calculating a distance-based coefficient of determination R^2^ estimated using an R package “vegan” [42]. The analysis was repeated for both unweighted and weighted UniFrac distance matrices separately for each condition and time point.

We further investigated the reproducibility of microbiome measurements across time stratified by condition. For each microbiome measurement, we first averaged technical replicates at time 0, 3 and 7 separately for each media condition and then calculated the ICC comparing the technical averages at time 3 vs. 0 and 7 vs. 0. Then, we tested whether the ICCs were similar across time and under different media conditions. Although we had specimens immediately frozen with no media as a control comparison, most of this analysis was based using the appropriate media frozen immediately after collection as the main control. Similarly, we quantified the overall temporal stability (comparing day 3 vs. day 0 and day 7 vs. day 0) by calculating R^2^ using weighted and unweighted UniFrac distance matrices for each condition. Although we had specimens immediately frozen with no media as a control comparison, most of this analysis was based using the appropriate media frozen immediately after collection as the main control. We tested whether the ICCs were different among the three media conditions using permutations. To test the association between ICC and relative abundance of the many taxa, a linear regression model was fitted to the log relative abundance of all taxa that had minimum relative abundance of 0.1 %.
